# Beyond experimentation: Five trajectories of cigarette smoking in a longitudinal sample of youth

**DOI:** 10.1371/journal.pone.0171808

**Published:** 2017-02-09

**Authors:** Lauren M. Dutra, Stanton A. Glantz, Nadra E. Lisha, Anna V. Song

**Affiliations:** 1 Center for Tobacco Control Research and Education, University of California, San Francisco, California, United States of America; 2 Center for Health Policy Science and Tobacco Research, RTI International, Berkeley, California, United States of America; 3 Department of Medicine, University of California, San Francisco, California, United States of America; 4 Psychological Sciences, Health Sciences Research Institute, University of California, Merced, California, United States of America; Dartmouth College Geisel School of Medicine, UNITED STATES

## Abstract

The first goal of this study was to identify the most appropriate measure of cigarette smoking for identifying unique smoking trajectories among adolescents; the second goal was to describe the resulting trajectories and their characteristics. Using 15 annual waves of smoking data in the National Longitudinal Survey of Youth 1997 (NLSY97), we conducted an exploratory latent class growth analysis to determine the best of four outcome variables for yearly smoking (cigarettes per day on days smoked, days smoked per month, mean cigarettes per day, and total cigarettes per month) among individuals aged 12 to 30 (n = 8,791). Days smoked per month was the best outcome variable for identifying unique longitudinal trajectories of smoking and characteristics of these trajectories that could be used to target different types of smokers for prevention and cessation. Objective statistics were used to identify four trajectories in addition to never smokers (34.1%): experimenters (13.6%), quitters (8.1%), early established smokers (39.0%), and late escalators (5.2%). We identified a quitter and late escalator class not identified in the only other comparable latent class growth analysis. Logistic regressions were used to identify the characteristics of individuals in each trajectory. Compared with never smokers, all trajectories except late escalators were less likely to be black; experimenters were more likely to be out of school and unemployed and drink alcohol in adolescence; quitters were more likely to have a mother with a high school degree/GED or higher (versus none) and to use substances in adolescence and less likely to have ever married as a young adult; early established smokers were more likely to have a mother with a high school diploma or GED, be out of school and unemployed, not live with both parents, have used substances, be depressed, and have peers who smoked in adolescence and to have children as young adults and less likely to be Hispanic and to have ever married as young adults; and late escalators were more likely to be Hispanic, drink alcohol, and break rules in adolescence and less likely to have ever married as young adults. Because of the number of waves of data analyzed, this analysis provided a clearer temporal depiction of smoking behavior and more easily distinguishable smoking trajectories than previous analyses. Tobacco control interventions need to move beyond youth-focused approaches to reach all smokers.

## Introduction

Tobacco-induced disease remains the leading preventable cause of death in the United States [1], with minorities bearing a disproportionate burden of the disease burden [2]. Because 80% of adult smokers begin smoking before age 18 [3], tobacco control efforts often focus on preventing adolescents from smoking their first cigarette. However, only one-third of youth who experiment with cigarettes ever become regular smokers [3,4]. As a result, these programs miss a key opportunity to prevent the transition from experimentation to established smoking, which may occur in the mid-to-late 20s [5]. In combination with growing recognition that young adulthood (ages 18 to 25) is a critical period of vulnerability [6], particularly due to significant life changes [3] such as starting college, separating smokers into different trajectories (patterns of smoking) and identifying when escalation and de-escalation occurs can inform efforts to prevent transition to regular smoking.

In addition to several regional U.S. latent class growth analyses of cigarette smoking dating back to the 1980s [4,7,8,9,10,11,12,13,14,15,16,17,18,19,20,21,22,23,24,25,26,27,28,29], three national studies of the four-wave longitudinal AddHealth (years 0, 1, 5–6, and 13–14) demonstrated that multiple trajectories of smoking behavior exist [30,31,32]. Two of the national analyses followed participants for the first three waves (to age 25) and identified five trajectories in addition to never smokers [30,32]. The third, by Fuemmeler et al. [31], used all four waves to track smoking behavior into young adulthood (age 32) and identified four trajectories, in addition to never smokers. These national analyses identified characteristics that distinguished between trajectories, including alcohol or drug use, deviance, maternal smoking, peer smoking, conduct problems, depressive symptoms, and state prevalence of adolescent smoking. (Covariates included gender, race/ethnicity, and parental education.) These studies did not assess school enrollment status, which is essential to determining which types of smokers are exposed to school-based prevention and cessation programs. These studies were limited to four waves, with multiple years between assessments. In addition, these studies only examined baseline predictors of membership in a given trajectory of smoking behavior. As a result, they did not identify characteristics of high-risk smokers that could be used to target them in young adulthood. The fact that they used two different measures of smoking (mean cigarettes per day and total cigarettes per month) does not allow a decision to be made about the best measure of smoking for assessing youth and young adult smoking behavior, which is typically lighter and more intermittent than among established adult smokers [33,34]. This paper reports a model building process using latent class growth analysis based on 15 years of annual data from the National Longitudinal Survey of Youth 1997 (NLSY97) to determine the best measure of smoking to identify distinct behavioral trajectories of smoking and differentiate between youth and young adults who progress to established smoking and those who quit smoking. By identifying these trajectories and the adolescent and young adult characteristics of those who follow them, this is the first analysis that can be used to create targeted national public health and clinical interventions for smoking prevention and early cessation that extend through young adulthood.

## Materials and methods

### Sample

The U.S. Bureau of Labor Statistics NLSY97 [35] began in 1997 with a sample of 8,984 youth aged 12 to 16 and provides 15 years of annual data on smoking (ages 26 to 30 at wave 15). The NLSY includes a nationally representative sample (n = 6,748) and an oversample of Hispanic/Latino and black respondents (n = 2,236) divided into five cohorts based on age (12 to 16) on December 31, 1996. Eighty-three percent (7,423 of 8,984) of those sampled in 1997 remained in 2011.

The analysis, which is based on the unrestricted public use NLSY97 dataset, included the 8,791 (98%) NLSY97 respondents with at least three non-missing years of data for 30-day cigarette use because a minimum of three points is necessary for latent class growth analysis (LCGA) [36]. All analyses used Bureau of Labor Statistics baseline weights [37] to adjust for NLSY sampling techniques, including the oversample of racial/ethnic minorities. This is an analysis of a de-identified dataset and was ruled exempt by the University of California San Francisco Committee on Human Research.

### Measures

#### Dependent variable: Smoking

Because there is no agreed upon measure of smoking for latent class growth analyses of youth and young adult smoking [30,31,32] and in order to determine the best distribution of the outcome data [38,39], we evaluated four smoking variables used in previous analyses: mean cigarettes smoked per day, cigarettes per day on the days smoked, days smoked per month, and total cigarettes smoked per month [4,8,15,30,31,40,41].

#### Independent variables

Race/ethnicity, gender, socioeconomic status (SES), and employment/school enrollment status at age 16 were examined as predictors of trajectory membership. Age 16 was chosen for childhood variables (such as employment/school enrollment status) because data were available for all respondents at this age and enrollment in school was mandatory. Race/ethnicity was coded as non-Hispanic white, non-Hispanic black, Hispanic, and non-Hispanic mixed race or non-Hispanic other. Gender was coded as male/female. Household poverty status at age 16 and highest level of biological mother’s education were used to measure childhood SES. Household poverty status was coded in relation to the previous year’s federal poverty line into four roughly equal categories (analyzed as continuous): below poverty line (0), poverty line to 199%, greater than poverty line (1), 200%–299% of the poverty line (2), and 300% or more of the poverty line (3). Biological mother’s highest education (assessed in 1997) was coded categorically as no degree (0), GED/high school degree (1), undergraduate degree (associate/junior college or bachelor) (2), and graduate school or above (masters, PhD, or professional degrees, such as MD or JD) (3). Employment/school enrollment status at age 16, included to reflect engagement, was categorically coded as enrolled in school but unemployed (0), employed but not enrolled in school (1), enrolled in school and employed (2), or neither employed nor enrolled in school (3). Age in 1997 was included in growth models to adjust for cohort effects [42].

We also tested several individual and interpersonal variables that could not be coded by age but were important to examine because they were included in previous LCGAs [30,31,32] of the AddHealth dataset. Depression in 2000 was coded continuously by taking the mean of responses to the five-question Mental Health Inventory adapted for use in the NLSY97 [43], with higher values reflecting higher levels of depression. The questions, which all had a four-point response scale ranging from “all of the time” (coded as 0) to “none of the time,” (coded as 3) included “How much of the time during the last month have you…” “been a nervous person,” “felt calm and peaceful,” “felt down or blue,” “been a happy person,” and “felt so down in the dumps that nothing could cheer you up?” Peer smoking in 1997, assessed by the question “What percentage of kids [in your grade/in your grade when you were last in school] [smoke/smoked] cigarettes?,” was coded continuously so that higher values of the variable reflected higher peer smoking using the five-point response scale that ranged from “almost none (less than 10%)” (coded as 0) to “almost all (more than 90%)” (coded as 4). We also measured conduct problems/rebellion in 2008, assessed using the question, “When I was in school, I used to break rules quite regularly,” as a continuous variable based on the 7-point response scale ranging from “disagreed strongly” (0) to “agree strongly” (6), with higher values reflecting a greater tendency for rule-breaking. We also assessed substance use in 1997 via dichotomized ever use of alcohol, marijuana, and “cocaine or other hard drugs” (reference category was never use). We assessed childhood family stability by dichotomizing responses to a question about whether the respondent lived with both biological parents in 1997, with a value of “1” signifying an individual who did not live with both biological parents in 1997 (and 0 as reference).

We examined several sociodemographic characteristics at age 26, the oldest available age for all respondents, to identify young adult characteristics of the different smoking trajectories. Marital status was ever married (married, separated, divorced, or widowed) versus never married (reference). Number of children was coded as one or more versus none (reference). Respondent’s highest level of education at age 26 was coded similarly to mother’s education.

### Analysis

LCGA is a person-centered approach to modeling that can be used to identify individuals with similar behavioral trajectories in adolescence and young adulthood [44]. Never smokers (34.1% of sample, n = 3,147), those who reported 0 days smoked in the past 30 days for all non-missing years of data collection, were specified as an *a priori* class and excluded from the LCGA [14]. This established approach [45] reduced the computational burden of the large number of zeros (from never smokers) and provided a better fit to the distribution.

We used M*plus* Version 7.3 because it accounts for the longitudinal structure of the NLSY and for missing data using full information maximum likelihood [46].

#### Determining the outcome variable

We first conducted a model building process to determine the best smoking variable to achieve the goal of LCGM, to produce “meaningful patterns that distinguish subgroups of people” [30] that could be targeted by public health interventions. We first constructed single-class LCGMs in order to make sure all models converged [47]. For mean cigarettes per day, we used a linear LCGM because the outcome was continuous. For all other measure of smoking, we used negative binomial LCGMs because they accommodate count variables and, unlike the Poisson distribution, account for unobserved heterogeneity in observations, leading to more accurate estimation of standard errors [48].

Because traditional fit statistics [49] were unavailable in Mplus because we used count variables [50] and had more than eight time points [51], to provide further guidance we examined the shape of the trajectories starting with a 4-class LCGM [31]. This model building process is an established method [4,8,15,31,47] to identify covariates, shapes of trajectories, and relationships between outcome and explanatory variables. [38,40,41]

#### Determining the number of trajectories

After we established the measure of smoking that provided the clearest differentiation between trajectories, a combination of theory and fit statistics was used to determine the ideal number of trajectories, starting with four [52]. The Bayesian Information Criteria (BIC), the Vuong-Lo-Mendell-Rubin Likelihood Ratio Test (LMR-LRT), the posterior probabilities, and the fraction of smokers in the trajectories were used to determine the most parsimonious model [47]. The BIC is a log-likelihood based statistic that accounts for number of model parameters, with lower values indicating better fit [52]. The LMR-LRT is a goodness-of-fit test that quantifies the likelihood that data can be described by a model with one less trajectory; a significant p-value (<0.05) indicates that the model with one additional trajectory has superior fit [52]. Respondents were assigned to the trajectory with the highest posterior probability, with probabilities above 0.7 considered acceptable [53]; all respondents met this criteria for trajectory assignment.

A cohort-sequential design [30] was used to link cohorts by age, resulting in 19 years (15 waves) of data (ages 12 to 30). This approach accounts for age-based cohort effects by designating age, not calendar year, as the time variable. We confirmed the validity of this approach by running separate LCGAs for each age group (12, 13, 14, 15, and 16 years old at baseline), which produced similar patterns of smoking at similar ages.

#### Determining predictors of trajectory membership

After the outcome variable and number of trajectories were determined and respondents were assigned to trajectories by *Mplus*, Holm-Sidak adjusted chi-square and t-tests and logistic regressions (never smokers were the reference group to make our results comparable to those of previous LCGAs of the AddHealth national samples [30,31,32]) were used to examine differences in participant characteristics across trajectories [54]. All analyses (bivariate tests and logistic regressions) were completed using Stata version 12 (StataCorp) with the svyset command to adjust for weights. Respondent’s highest education was not included in adjusted models because of collinearity with employment/school enrollment status and mother’s education. We included youth SES measures (income and mother’s education) and educational engagement (employment/school enrollment status) because these factors are more stable over time than adult income and education. The final model, which included age in 1997 to adjust for age-cohort effects, included all predictor variables that were significant in bivariate analyses. We also examined prevalence of daily smoking by trajectory, median years of daily smoking, and median years between first reported smoking and daily smoking (adjusted for weights) to further understand patterns of smoking in each trajectory.

## Results

All of the univariate latent class growth models converged except for mean cigarettes per day; therefore, this outcome was discarded. We then compared the trajectories of the other three outcome variables (S1 Table). We chose days per month as our outcome variable because it produced the most unique trajectories and a visible differentiation between smokers who progressed to daily smoking versus those who quit [30]. Days smoked per month is also a preferable measure of smoking intensity for youth and young adults because this variable provided unique patterns of smoking behavior and because cigarettes per day can be unstable over time [55]. Choosing this variable also achieved our aim of capturing progression (or lack thereof) to regular smoking and intermittent and nondaily smoking, which is a common trend among adolescents and young adults [33,34].

### Overall sample characteristics

Approximately half of the sample was female (48.7%) and was primarily non-Hispanic white (66.6%) (Table 1). About half the respondents (52.9%) had mothers whose highest education was GED/high school diploma. At age 16, 88.6% were in school, including 54.7%) who were employed while attending school. Mean household income was about twice (mean = 192%, median = 200%) the poverty level. In 1997, mean peer smoking was 1.63 (on average, participants estimated that approximately between 25% and 50% of their peers smoked), mean likelihood of breaking rules in school was 2.26 (between “disagree a little” and “neither agree nor disagree”), 47.3% did not live with both biological parents in 1997, 45.1% had ever drunk alcohol, 21.0% had ever tried marijuana, and 7.1% had ever used cocaine or hard drugs in 1997. Mean depression score in 2000 (range from 0 to 4) was 0.93 (symptoms of depression “some of the time”).

**Table 1:** Individual-level characteristics by trajectory of cigarette smoking.

Characteristic	Overall sample	Never smokers (trajectory 0)	Experimenters (trajectory 1)	Quitters (trajectory 2)	Early established smokers (trajectory 3)	Late escalators (trajectory 4)
	(N = 8,791)	34.1% (n = 3,147)	13.6% (n = 1,205)	8.1% (n = 701)	39.0% (n = 3,205)	5.2% (n = 533)
	%	%	%	%	%	%
	Mean (95%CI)	Mean (95%CI)	Mean (95%CI)	Mean (95%CI)	Mean (95%CI)	Mean (95%CI)
**Youth variables (1997 unless otherwise noted)**						
**Female**	51.3%	53.1%^a^	53.2%^a^	47.6%^a,b,c^	44.0%^b^	45.6%^c^
**Race/ethnicity**						
• Non-Hispanic white	66.6%	61.7%^a^	66.6%^b^	70.3%^b^	72.5%^c^	49.3%^d^
• Non-Hispanic black	15.6%	19.3%	12.0%	11.8%	13.1%	24.7%
• Hispanic	13.0%	13.6%	17.2%	14.2%	9.7%	20.0%
• Non-Hispanic Mixed/other	4.9%	0.5%	4.2%	3.7%	4.7%	6.2%
**Mother’s highest education**						
1. <GED/HS diploma	12.6%	11.6%^a,b^	11.2%^a^	11.2%^a,b^	13.7%^b,c^	15.7%^b,c^
2. GED/HS graduate	52.9%	52.0%	48.9%	53.0%	55.7%	48.4%
3. AA/BA/BS	27.4%	28.2%	29.4%	30.0%	24.9%	31.1%
4. Graduate/ professional Degree	7.2%	8.1%	10.6%	6.0%	5.7%	4.9%
**Employment/school enrollment status (age 16)**						
• In school, not employed	33.9%	37.0%^a,d^	33.6%^a,b^	31.1%^b^	30.5%^c^	43.8%^d^
• Employed, not in school	6.1%	3.4%	3.7%	5.0%	9.8%	4.0%
• Employed and in school	54.7%	56.9%	59.7%	57.9%	50.9%	49.5%
• Neither in school nor employed	5.4%	2.7%	3.1%	5.9%	8.8%	2.7%
**Household income (age 16)**	1.92 (1.87–1.96)	2.06 (2.02–2.10)^a^	2.19 (2.13–2.26)^a^	2.20 (2.12–2.28)^a^	1.95 (1.91–1.98)^b^	2.00 (1.90–2.10)^a,b^
**Depression (2000)**	0.93 (0.92–0.95)	0.85 (0.83–0.87)^a^	0.95 (0.92–0.98)^b^	0.98 (0.94–1.02)^b,c^	1.00 (0.98–1.02)^c^	0.92 (0.87–0.97)^b^
**Peer smoking**	1.63 (1.60–1.65)	1.36 (1.31–1.41)^a^	1.52 (1.45–1.60)^b^	1.74 (1.64–1.84)^c^	1.88 (1.84–1.93)^d^	1.49 (1.37–1.62)^e^
**Broke rules in school (2008, retrospective)**	2.26 (2.20–2.31)	1.43 (1.35–1.50)^a^	1.95 (1.83–2.08)^b,e^	2.53 (2.36–2.71)^c^	3.02 (2.92–3.11)^d^	2.19 (1.99–2.39)^e^
**Non-two-parent family**	47.4%	41.6%^a^	56.7%^a^	44.2%^a^	38.6%^b^	43.7%^a^
**Ever drank alcohol**	45.1%	26.8%^a^	59.0%^b^	56.4%^c^	46.3%^c^	41.4%^b^
**Ever used marijuana**	21.0%	6.6%^a^	34.7%^b,e^	28.9%^c^	16.1%^e^	13.0%^e^
**Ever used cocaine/hard drugs (1998)**	7.1%	2.0%^a^	11.9%^b^	12.3%^c^	4.8%^c^	2.8%^a,b^
**Adult variables (age 26)**						
**Ever married**	40.7%	44.7%^a^	44.4%^a^	48.1%^a^	35.9%^b^	30.0%^c^
**Has ≥1 child**	45.6%	40.7%^a^	39.0%^a,c^	44.7%^a,c^	53.1%^b^	39.8%^c^
**Respondent’s highest education**						
1. <GED/HS diploma	9.4%	4.0%^a^	5.0%^a^	7.5%^b^	15.9%^c^	8.2%^b^
2. GED/HS graduate	55.3%	45.8%	45.9%	55.3%	66.4%	56.9%
3. AA/BA/BS	30.6%	42.1%	39.2%	32.1%	16.6%	31.2%
4. Graduate/ professional degree	4.7%	8.1%	6.1%	5.2%	1.2%	3.6%

Notes: Household income coded as an ordinal variable according to the poverty level the year before data collection (0 = below poverty level, 1 = poverty level to 199% of poverty level, 2 = 200%–299% of poverty level, 3 = 300% or more of poverty level)

Matching superscripts indicate no differences between groups.

We used chi-squares and t-tests to compare trajectories for categorical and continuous variables, respectively

Family error rate (.05) for multiple comparisons using the Holm-Sidak adjustment

By age 26, most of the respondents (59.3%) had never been married and had no children (54.4%). At age 26, 9.4% had no degree, 55.3% had a GED/high school diploma, 30.6% had a college degree, and 4.7% had a graduate or professional degree.

### Trajectories of smoking from 1997–2011

We tested one to five trajectories. The BIC consistently declined across solutions (S2 Table). The LMR-LRT p-value increased across trajectories, reaching non-significance at five trajectories. Since this result suggested five trajectories was not superior to four and all posterior probabilities were over 0.7 for all solutions (0.90–0.98), we selected four classes.

**Fig 1:**
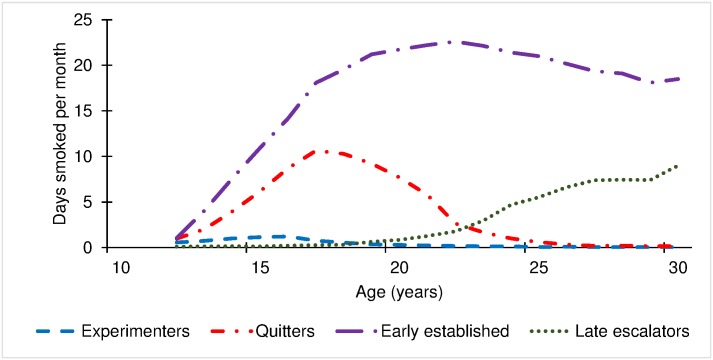
Four trajectories of smoking behavior among ever smokers in the National Longitudinal Survey of Youth 1997 (NLSY97). Analyses of 19 years (15 waves) of smoking data (days smoked per month) for participants in the NLSY97 revealed four patterns of smoking behavior (in addition to never smokers): experimenters, quitters, early established smokers, and late escalators.

Based on trajectory shapes and the literature, we named the four trajectories (in addition to never smokers, trajectory 0) experimenters (trajectory 1), quitters (trajectory 2), early established smokers (trajectory 3), and late escalators (trajectory 4) (Fig 1). Never smokers comprised 34.1% (n = 3,147) of the sample, experimenters 13.6% (n = 1,205), quitters 8.1% (n = 701), early established smokers 39.0% (n = 3,205), and late escalators 5.2% (n = 533).

Days smoked per month peaked at age 16 for experimenters (mean = 1.22; 95% CI: 0.96–1.48), with mean days smoked per month remaining below one for 16 of the 19 years. For quitters, days smoked peaked at age 17 (10.64; 9.61–11.67) and decreased to less than one day per month at age 25. For early established smokers, days smoked peaked at age 22 (22.60; 22.14–23.05) and plateaued by age 30 (18.49; 17.21–19.78). Late escalators’ highest smoking was 9.00 (6.11–11.89) days per month at age 30 and still trending upward.

Only 2.0% of experimenters, 49.5% of quitters, 93.5% of early established smokers, and 24.0% of late escalators ever smoked daily. Median years of daily smoking was 0 for experimenters (10th–90th percentile: 0–0; only 2.0% ever smoked daily), 0 for quitters (0–3), 6 for early established smokers (1–12), and 0 for late escalators (0–2). Median years from first cigarette to daily smoking was 0 (0–1) for the 2.0% (23 of 1,205) of experimenters who made this transition, 1 year (0–4) for the 48.4% (339 of 701) of quitters who made this transition, 1 year (0–5) for the 92.5% (2,966 of 3,205) of early established smokers who made this transition, and 3 years (0–11) for the 26.3% (140 of 533) of late escalators who made this transition. Median age of transition to daily smoking (for those who transitioned) was 15 (13–16) for experimenters, 17 (15–20) for quitters, 17 (14–21) for early established smokers, and 26 (24–28) for late escalators.

### Predictors of trajectory membership

Our adjusted model included all covariates (Table 2). Compared with never smokers, experimenters were more likely to have ever tried alcohol in 1997 and less likely to be non-Hispanic black (than non-Hispanic white) and to be employed and enrolled in school at age 16 (versus not enrolled in school and unemployed). Quitters were more likely to be unemployed at age 16, to have a mother who had at least completed high school/GED, and to have ever drank alcohol or used marijuana in 1997. They were less likely to be black, to be enrolled in school at age 16, and to have ever been married by age 26. Early established smokers were more likely to have a mother whose highest level of education was a GED/high school diploma (versus no degree), to have used alcohol or marijuana in 1997, have peers who smoked in 1997, to be depressed in 2000, have broken rules in school in 2008, and to have children at age 26. They were less likely to be black or Hispanic, to be enrolled in school or employed at age 16, to live with both biological parents in 1997, and to have ever been married at age 26. Late escalators were more likely to be Hispanic, to have drank alcohol in 1997, and to have broken rules in school and less likely to have ever been married at age 26 than never smokers.

**Table 2:** Adjusted odds of trajectory membership for all significant covariates (with 95% confidence intervals).

	Experimenters (trajectory 1) versus never smokers (trajectory 0)	Quitters (trajectory 2) versus never smokers (trajectory 0)	Early established smokers (trajectory 3) versus never smokers (trajectory 0)	Late escalators (trajectory 4) versus never smokers (trajectory 0)
**Youth variables (baseline unless otherwise noted)**				
**Gender**				
• Female	REF	REF	REF	REF
• Male	1.20 (0.72–2.00)	1.01 (0.55–1.84)	1.40 (0.95–2.07)	1.06 (0.53–2.11)
**Race/ethnicity**				
• Non-Hispanic white	REF	REF	REF	REF
• Non-Hispanic black	0.45 (0.24–0.82)	0.19 (0.09–0.40)	0.28 (0.18–0.45)	0.80 (0.37–1.75)
• Hispanic	1.61 (0.87–2.99)	0.77 (0.33–1.81)	0.40 (0.24–0.69)	2.62 (1.15–6.01)
• Non-Hispanic mixed/other	1.36 (0.43–4.31)	0.32 (0.05–1.92)	1.04 (0.38–2.82)	1.00 (empty)
**Mother’s highest education**				
1. <GED/HS diploma	REF	REF	REF	REF
2. GED/HS graduate	1.69 (0.91–3.16)	3.29 (1.41–7.65)	1.92 (1.15–3.20)	2.04 (0.89–4.68)
3. AA/BA/BS	1.62 (0.78–3.36)	2.92 (1.07–7.91)	1.55 (0.83–2.86)	2.73 (0.99–7.54)
4. Graduate/ professional degree	2.07 (0.71–6.05)	4.24 (1.09–16.43)	0.83 (0.33–2.11)	2.19 (0.39–12.24)
**Employment/ school enrollment status (age 16)**				
• In school, not employed	REF	REF	REF	REF
• Employed, not in school	0.97 (0.43–2.19)	0.92 (0.35–2.40)	1.63 (0.85–3.13)	0.80 (0.25–2.59)
• Employed and in school	1.01 (0.55–1.85)	0.70 (0.34–1.45)	0.65 (0.41–1.02)	0.88 (0.44–1.76)
• Neither in school nor employed	2.52 (1.09–5.83)	2.80 (0.98–8.02)	4.34 (2.25–8.37)	0.77 (0.24–2.52)
**Non-two-parent family**	0.78 (0.49–1.26)	1.38 (0.77–2.47)	1.65 (1.12–2.42)	1.01 (0.51–1.97)
**Ever drank alcohol**	2.50 (1.51–4.14)	3.08 (1.58–6.01)	2.53 (1.65–3.89)	2.21 (1.09–4.47)
**Ever used marijuana**	1.19 (0.63–2.23)	3.45 (1.70–6.99)	2.11 (2.07–5.53)	0.74 (0.28–1.97)
**Ever used cocaine/hard drugs (1998)**	0.80 (0.24–2.62)	1.01 (0.34–2.96)	1.57 (0.68–3.58)	1.00 (empty)
**Household income (age 16)**	1.30 (0.99–1.71)	0.93 (0.66–1.30)	0.85 (0.68–1.06)	0.81 (0.55–1.17)
**Age**	0.78 (0.63–0.95)	0.79 (0.62–1.01)	0.85 (0.73–1.001)	0.97 (0.72–1.32)
**Depression (2000)**	1.17 (0.76–1.82)	1.45 (0.85–2.47)	1.72 (1.27–2.61)	1.21 (0.63–2.32)
**Peer smoking**	1.19 (0.98–1.46)	1.19 (0.92–1.53)	1.26 (1.08–1.48)	0.98 (0.75–1.27)
**Likely to have broken rules in school (2008, retrospective)**	1.06 (0.94–1.19)	1.15 (0.99–1.33)	1.28 (1.17–1.41)	1.26 (1.07–1.47)
**Adult variables (age 26)**				
**Marital status**				
• Never married	REF	REF	REF	REF
• Ever Married	0.78 (0.49–1.29)	0.52 (0.28–0.94)	0.47 (0.31–0.70)	0.42 (0.22–0.81)
**Has ≥1 child**				
• No	REF	REF	REF	REF
• Yes	1.28 (0.77–2.13)	1.12 (0.59–2.15)	1.76 (1.15–2.68)	1.10 (0.58–2.06)

Notes: All models include all listed variables.

All models adjusted for stratification variables (to adjust for sampling techniques) and weights (to adjust for non-response and non-representativeness of sample) provided by the NLSY

All of the variance inflation factors were 1.44 or under in the final adjusted model, indicating little multicollinearity. The results of the unadjusted analyses (see Table 1) were similar to the adjusted results (see Table 2). The only variables that were significant in bivariate analyses but not significant in adjusted models were household income, which was significantly lower for early established smokers than members of the other trajectories (except late escalators), and ever use of cocaine/hard drug use, which was more common for experimenters and quitters than other trajectories.

## Discussion

Exploratory analyses revealed that days smoked per month provided clearer distinction between trajectories of smoking than cigarettes per day on days smoked, mean cigarettes per day, or total cigarettes smoked per month. LCGA revealed four trajectories in addition to never smokers: experimenters, quitters, early established smokers, and late escalators. This finding contradicts the assumption that once adolescents initiate smoking, they all exhibit the same pattern of smoking: 21.4% of ever smokers were experimenters who never went on to become established smokers.

### Trajectory characteristics

We found several qualitative and quantitative differences between our trajectories and those identified in LCGAs of AddHealth (S3 and S4 Tables) [30,31,32]. Fuemmeler et al. [31] did not identify a quitter class; this group is key to understanding why some individuals spontaneously quit. In addition, neither Fuemmeler et al. [31] nor Pollard et al. [32] identified a true late escalator class (started smoking after age 20 and trending upward at end of follow-up), thereby failing to identify an important minority group that has been ignored in the past. Fuemmeler et al. [31] and Pollard et al.’s [32] experimenter classes peaked much later (ages 24 and 25, respectively) than ours (age 16). Costello et al.’s [30] quitter class peaked at age 20, later than our quitter class, which peaked at age 16.

Some of these differences may be due to our specification of never smokers as an a priori class (as opposed to including never smokers in the LCGA). As a result, our never smoker category was smaller than those identified in analyses of AddHealth (S4 Table). In addition, our use of days smoked per month (versus cigarettes per day or a combined measure of frequency and intensity) distinguishes our analysis from those completed with AddHealth. Most importantly, the great number of waves of data collection in the NLSY97 (annually between 1997 and 2011) resulted in a level of detail unavailable when using AddHealth’s three or four waves of data (1995, 1996, 2001–2002, 2008–2009). This important difference enabled us to identify specific ages at which key transitions occur.

For example, *early established* smokers escalated between age 12 and 22, *late escalators* escalated between age 22 and 30, and *quitters* de-escalated between 18 and 24. As a result, we concluded that late escalators should be targeted in young adulthood. In the Fuemmeler et al. [31] analysis, in particular, with the exception of the late heavy user group, the most distinguishing graphical characteristic between the classes was the intercept. In contrast, our analysis revealed trajectories with differing intercepts, slopes, and shapes.

Like the current analysis and analyses of Add Health [30,31,32], LCGAs of smoking of the sub-national samples [4,7,8,9,10,11,12,13,14,15,16,17,18,19,20,21,22,23,24,25,26,27,28,29] provides support for the existence of multiple smoking trajectories. The findings of the sub-national studies, however, are less likely to be generalizable to the entire United States, so have limited applicability to the development of national policies and interventions for smoking prevention and cessation.

### Characteristics of individuals in trajectories

Analyses of AddHealth [30,31,32] revealed that alcohol or drug use, deviance, maternal smoking, peer smoking, conduct problems, depressive symptoms, and state prevalence of adolescent smoking distinguished one or more smoking trajectories from never smokers. With the exception of state smoking prevalence, which we did not include in our analysis, our results demonstrated similar findings for these variables.

We also identified several characteristics that were not identified in previous analyses (S4 Table). Members of all trajectories except experimenters were less likely to have been married at age 26 compared with never smokers. Compared with never smokers, *experimenters* were less likely to be black and less likely to be enrolled in school or working at age 16. However, overall, experimenters were similar to never smokers. Our results suggest that tobacco control programs should expand their efforts beyond preventing experimentation to also preventing escalation to established smoking.

*Quitters* were less likely to be black, tended to have mothers with higher education, and were more likely to have ever used marijuana. Compared with early established smokers, quitters were more likely to be Hispanic and less likely to break rules in school. Additional research should identify further characteristics about this group and their reasons for quitting. However, the existence of this group likely indicates that traditional youth-based smoking prevention and cessation programs are successful for some adolescents.

*Early established* smokers were less likely to be black or Hispanic, to have educated mothers, and to grow up in a two-parent household; they were more likely to have ever used marijuana and to drop out of school by age 16. As a result, they are probably less likely to be reached by school-based interventions in adolescence.

*Late escalators* only exhibited some traditional smoking risk factors, were more likely to be Hispanic, and were still enrolled in school at age 16. Because their smoking escalates after age 18, they are more likely to benefit from interventions that extend into young adulthood.

### Public health policy implications

This is the first analysis that can be used to create targeted national public health and clinical interventions for smoking prevention and early cessation that extend through young adulthood. Population-level interventions, including tobacco taxes, smokefree laws (particularly workplace) and rules (such as car), smokefree housing, and media campaigns are key to reaching all types of smokers [56,57,58]. People covered by smokefree laws are more likely to have smokefree cars [56] and homes [59], which increase the likelihood of successful smoking cessation [57,60]. In addition, as recommended by the American Academy of Pediatrics [61], all movies and video games depicting smoking should be given an R rating [62,63] or eliminated entirely. Similarly, restricting *all* tobacco advertising, including point-of-sale and product placement, would deter smoking initiation and adolescent smoking and progression to established smoking. In addition to these approaches, we identified specific methods of reaching early established smokers and late escalators. Because early established smokers are more likely to have children, one method of reaching them is by incorporating smoking cessation interventions into early intervention programs, such as Women, Infants, and Children, an assistance program for pregnant women and children under age 5, and California’s “First Five,” a county-based early intervention program [40], which is funded by tobacco taxes. To reach late escalators in young adulthood when their smoking frequency is increasing, we recommend smokefree schools, smokefree college campuses, college-based smoking cessation programs, and bar-based smoking cessation and prevention interventions [64].

The Truth Initiative, which targets youth and young adults [65], and the Centers for Disease Control and Prevention’s *Tips From Former Smokers* media campaign, which targets 18- to 54-year-olds [66], are likely to be effective for early established smokers and late escalators. Media campaigns, such as the Food and Drug Administration’s *The Real Cost* and *Fresh Empire* campaigns [41,67], should be extended to include individuals into their late 20s, particularly since youth-centric programs are an industry tactic [68].

Because of the immense gains made in tobacco control in the past 50 years, including denormalizing tobacco and revolutionizing social norms, it is necessary to reevaluate the efficacy of the established approach to tobacco prevention, particularly as new databases emerge. The Population Assessment of Tobacco and Health (PATH) study should be used to continue to identify patterns of smoking to prevent progression to established smoking and encourage smoking cessation and identify the effects of rising dual use of tobacco products (including e-cigarettes) and marijuana on trajectories of cigarette smoking.

### Limitations

The primary limitation of this analysis is that it does not include policy variables that affect smoking. It is possible that individuals shift trajectories over time, but LCGAs do not accommodate such shifts.

### Conclusion

Days smoked per month is a useful measure of smoking for identifying different types of smokers and should therefore be included in tobacco use surveys. Also, multiple patterns of smoking are necessary to fully describe adolescent and young adult smoking uptake and patterns of use. Just as the tobacco industry identified different types of smokers (market segments) to target, tobacco control programs can use information about types of smokers to develop more effective interventions [69]. Targeting young adults (18 to 24) is important because this group is most likely to quit and do so successfully. Pediatricians should collect information on smoking intensity and frequency and attempt to prevent smoking escalation among light smokers by helping them identify triggers, referring them to counseling resources, and encouraging youth to advocate a smokefree lifestyle for parents and peers. Because smoking is still dynamic beyond age 18, pediatricians and other physicians should ensure that information about tobacco use is transferred to general medicine practitioners as their patients get older. Our analysis also supports increasing the smoking age from 18 to 21 [61]; 16.9% (954) of the ever smokers in our analysis initiated smoking between the ages of 18 and 21.

## Supporting information

S1 Table Comparison of four-class solutions by smoking variable. (DOCX)

S2 Table Fit statistics and sample size (N) by number of classes. (DOCX)

S3 Table Graphical comparison of trajectories from other latent class growth analyses using national samples. (DOCX)

S4 Table Comparison of this paper with comparable trajectories identified in previous latent class growth analyses of national datasets. (DOCX)

S1 Data Raw data file as stata 12 DTA file. (DTA)
