# Supplementary material for: Beyond experimentation: Five trajectories of cigarette smoking in a longitudinal sample of youth
Source: PLoS One. 2017 Feb 9;12(2):e0171808. doi: 10.1371/journal.pone.0171808 (PMC5300123; doi:10.1371/journal.pone.0171808)
Supplement: S1 Table — (DOCX) [file pone.0171808.s001.docx]

| **S1 Table. Comparison of four-class solutions by smoking variable** | | | | | |
| --- | --- | --- | --- | --- | --- |
|  |  | Percent of smokers in each class | | | |
| Variable |  | Class 1 | Class 2 | Class 3 | Class 4 |
| Cigarettes per day on days smoked |  | 21.7% | 25.7% | 27.9% | 24.7% |
| Days smoked out of past 30 |  | 8.1% | 12.9% | 20.0% | 59.0% |
| Total cigarettes per month |  | 8.5% | 14.5% | 20.6% | 56.4% |
