# Supplementary material for: Beyond experimentation: Five trajectories of cigarette smoking in a longitudinal sample of youth
Source: PLoS One. 2017 Feb 9;12(2):e0171808. doi: 10.1371/journal.pone.0171808 (PMC5300123; doi:10.1371/journal.pone.0171808)
Supplement: S2 Table — (DOCX) [file pone.0171808.s002.docx]

| **S2 Table. Fit statistics and sample size (N) by number of classes** | | | | | | | | | | | |
| --- | --- | --- | --- | --- | --- | --- | --- | --- | --- | --- | --- |
|  | | | Sample size by class | | | | | | | |  |
| Classes | BIC^a^ | Entropy | N1 | N2 | N3 | N4 | N5 | N6 | N7 | N8 | LMR^b^ |
| 1 | 491526.655 | - | 8,791 | - | - | - | - | - | - | - | - |
| 2 | 461068.978 | 0.963 | 4,349 | 2,566 | - | - | - | - | - | - | 0.000 |
| 3 | 454598.884 | 0.930 | 1,125 | 2,203 | 3,587 | - | - | - | - | - | 0.000 |
| 4 | 435517.578 | 0.930 | 3,344 | 693 | 1,161 | 445 | - | - | - | - | 0.004 |
| 5 | 434366.800 | 0.908 | 564 | 1,052 | 3,142 | 472 | 414 | - | - | - | 0.322 |
| ^a^ BIC: Bayesian Information Criterion  ^b^ LMR: Lo-Mendell Rubenstein Adjusted Least Likelihood Ratio Test p-value | | | | | | | | | | | |
