# Supplementary material for: Beyond experimentation: Five trajectories of cigarette smoking in a longitudinal sample of youth
Source: PLoS One. 2017 Feb 9;12(2):e0171808. doi: 10.1371/journal.pone.0171808 (PMC5300123; doi:10.1371/journal.pone.0171808)
Supplement: S3 Table — (DOCX) [file pone.0171808.s003.docx]

| **S3 Table. Graphical comparison of trajectories from other latent class growth analyses using national samples** | |
| --- | --- |
| This paper: NLSY97 |  |
| Fuemmeler et al [31] (2013): AddHealth | 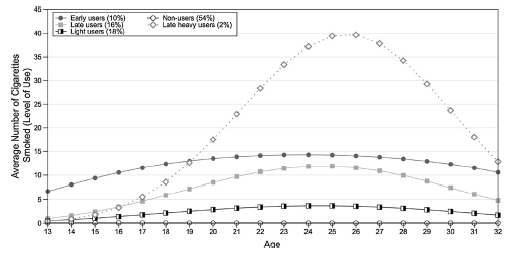 |
| Pollard et al [32] (2010): AddHealth | 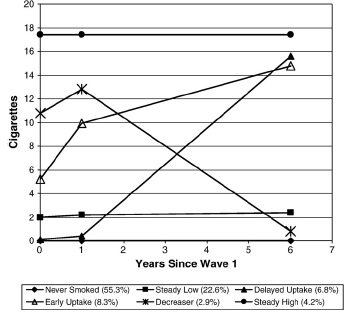 |
| Costello et al [45] (2008): AddHealth | 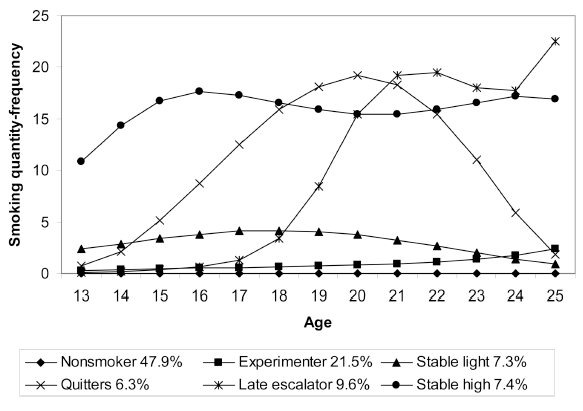 |
