# Supplementary material for: Beyond experimentation: Five trajectories of cigarette smoking in a longitudinal sample of youth
Source: PLoS One. 2017 Feb 9;12(2):e0171808. doi: 10.1371/journal.pone.0171808 (PMC5300123; doi:10.1371/journal.pone.0171808)
Supplement: S4 Table — (DOCX) [file pone.0171808.s004.docx]

| **S4 Table. Comparison of this paper with comparable trajectories identified in previous latent class growth analyses of national datasets** | | | | |
| --- | --- | --- | --- | --- |
|  | **This paper** | **Fuemmeler et al [31] (2013)** | **Pollard et al [32] (2010)** | **Costello et al [45] (2008)** |
| **Trajectory names from this paper** | % of sample, description | % of sample, qualitative differences from this paper, original trajectory name | | |
| **Never smokers** | 34% | 54%, Non-users | 55%, Never smoked | 48%, Nonsmoker |
| **Experimenter** | 14%, <1 day at age 12, peaked at 16, <1 day by 17 | 18%, peaked at 24, light users | 23%, did not peak/ straight line, light users | 22%, peaked at 25, experimenters |
| **Quitter** | 8%, 1 day at age 12, peaked at age 17, <1 day by age 25 (8%) | Not identified | 3%, similar, decreaser | 6%, peaked at age 20, quitters |
| **Early established** | 39%, 1 day at age 12, peak at 22, plateau at 30 at >1 day | 10%, smaller slope, early users | 8%, similar, early uptake | 7%, intercept higher, peaked at age 16, stable high |
| **Late escalators** | 5%, <1 day at 12, >1 at age 21, peaked at age 30 at >1 day | Not identified | 7%, >1 by 14-21, peaked at 18-25, delayed uptake | 10%, >1 by age 17, peaked at age 25, late escalators |
|  | Not identified | 16%, close to 0 at 13, peaked at 25, >0 at age 32, late users | Not identified | Not identified |
|  | Not identified | 2%, close to 0 at 13, peaked at 26, >0 at age 32, late heavy users | 4%, >1 cig at year 0, did not peak/straight line, >1 cig at year 6, steady high | 7%, >1 at age 13, peaked at age 17, <1 at age 25, stable light |
| **Methods** | | | | |
| **Dataset** | NLSY97 | AddHealth | | |
| **Waves of data** | 15 (annual) | 4 (years 0, 1, 5-6, 13-14) | 3 (years 0, 1, 5-6) | 3 (years 0, 1, 5-6) |
| **Smoking measure** | Days smoked per month | Mean cigs smoked/day on days smoked | Mean cigarettes per day | Categorical frequency x categorical intensity |
